# Evidence that an HLA-DQA1-DQB1 haplotype influences susceptibility to childhood common acute lymphoblastic leukaemia in boys provides further support for an infection-related aetiology.

**DOI:** 10.1038/bjc.1998.540

**Published:** 1998-09

**Authors:** G. M. Taylor, S. Dearden, N. Payne, M. Ayres, D. A. Gokhale, J. M. Birch, V. Blair, R. F. Stevens, A. M. Will, O. B. Eden

**Affiliations:** Immunogenetics Laboratory, St Mary's Hospital, Manchester, UK.

## Abstract

Comparison of DQA1 and DQB1 alleles in 60 children with common acute lymphoblastic leukaemia (c-ALL) and 78 newborn infant control subjects revealed that male but not female patients had a higher frequency of DQA1*0101/*0104 and DQB1*0501 than appropriate control subjects. The results suggest a male-associated susceptibility haplotype in c-ALL and supports an infectious aetiology.


British Journal of Cancer (1998) 78(5), 561–565

© 1998 Cancer Research Campaign

### Evidence that an *HLA-DQA1–DQB1* haplotype influences susceptibility to childhood common acute lymphoblastic leukaemia in boys provides further support for an infection-related aetiology

**GM Taylor[1], S Dearden[1], N Payne[1], M Ayres[1], DA Gokhale[1], JM Birch[2], V Blair[2], RF Stevens[3], AM Will[3] and OB Eden[4]**

[1]Immunogenetics Laboratory, St Mary's Hospital, Manchester M13 0JH, UK; [2]CRC Paediatric and Familial Cancer Research Group and [3]Department of Haematology, Royal Manchester Children's Hospital; [4]Academic Department of Paediatric Oncology, Christie Hospital and Royal Manchester Children's Hospital, Manchester, UK

**Summary** Comparison of *DQA1* and *DQB1* alleles in 60 children with common acute lymphoblastic leukaemia (c-ALL) and 78 newborn infant control subjects revealed that male but not female patients had a higher frequency of *DQA1*0101/*0104 and DQB1*0501* than appropriate control subjects. The results suggest a male-associated susceptibility haplotype in c-ALL and supports an infectious aetiology.

**Keywords:** childhood common acute lymphoblastic leukaemia; *HLA-DQA1*; *DQB1*; aetiology; genetic susceptibility; infection

Evidence suggesting that childhood common acute lymphoblastic leukaemia (c-ALL) may have an infectious aetiology (reviewed by Greaves, 1997) continues to accumulate. Associations with socioeconomic status (Alexander et al, 1990), time–space case clustering (Alexander, 1992), population isolation and mixing (reviewed by Kinlen, 1995), parental occupation (Kinlen, 1997) and seasonality (Badrinath et al, 1997; Westerbeek et al 1998) all point to factors affecting the transmission of an infectious agent. However, no leukaemogenic infectious agent has yet been identified in c-ALL.

Results suggesting that infectious diseases may be no more common in children before the development of ALL than they are in normal children (van Steensel-Moll et al, 1986) imply that the immune response may itself contribute to the aetiology of c-ALL (Greaves, 1988; Greaves and Alexander, 1993). If differences in the efficiency of such immune responses do influence the risk of c-ALL, this may be related to the differential antigen-presenting capacity of HLA class II alleles. We previously showed that children with c-ALL typed more frequently for *HLA-DQB1*0501* than control subjects (Dearden et al, 1996). As *DQB1* is very tightly linked to *DQA1*, we carried out a molecular analysis of *DQA1* alleles in patients previously typed for *DQB1* to see if both genes influenced susceptibility to c-ALL.

### MATERIALS AND METHODS

### Patients and control subjects

The patients consisted of an unselected series of 60 children (38 boys, 22 girls) with c-ALL from the same series as described previously (Dearden et al, 1996). Control blood samples were obtained from the umbilical cords of 78 normal full-term newborn infants (38 boys and 40 girls) delivered at St Marys Hospital.

### *HLA-DQA1* molecular typing

Genomic DNA was extracted from patient and control blood samples as previously described (Dearden et al, 1996), and *DQA1* typing carried out as described by Noreen et al (1992). A 228-bp exon 2 fragment of *DQA1* was amplified by the polymerase chain reaction (PCR) using the primers DQAAMP-A (5′-ATG GTG TAA ACT ACC AGT-3′) and DQAAMP-B (5′-TTG GTA GCA GCG GTA GAG TTG-3′), obtained from the British Society for Histocompatibility and Immunogenetics (BSHI). PCR mixtures consisted of 50 ng of genomic DNA, 0.5 μM of each primer and 0.3 mM dNTPs in 20 μl of PCR buffer. Amplifications (32 cycles) were carried out on a Thermal Cycler (Appligene, France), and PCR products dot-blotted onto nylon filters, which were hybridized with ten 18-mer sequence-specific oligonucleotide (SSO) probes (from BSHI) end-labelled with $\gamma^{32}$P-ATP, detecting eight *DQA1* alleles [**0101 (*0101 + *0104)*, **0102*, **0103*, **0201*, **03 (*03011 + *0302)*, **0401*, **05 (*05011 + *0502 + *0503)* and **0601*]. The filters were scanned for radioactivity on an InstantImager (Canberra Packard, Berks, UK). Positive hybridization was scored by comparing test vs negative control spots (typically a tenfold difference in counts). *DQA1* alleles were assigned by comparing SSO patterns with published information (see Marsh reference to Website). The following pairs of alleles could not be distinguished from each other with the probes used in this study: **0101* from **0104*; **03011* from **0302* and subtypes of **05*; **0601* homozygotes gave the same result as **0401/*0601* heterozygotes; and **0103* homozygotes gave the same result as **0102/*0103* heterozygotes.

### *DQB1* molecular typing

Patients and control subjects were typed for *DQB1* alleles by single-strand conformation polymorphism (SSCP) analysis as described previously (Dearden et al, 1996).

*Received 28 November 1997*
*Revised 16 March 1998*
*Accepted 17 March 1998*

*Correspondence to:* GM Taylor

**Table 1** *HLA-DQA1* allele frequencies in childhood common ALL compared with infant control subjects

| Allele group | *DQA1* allele[a] | Total | | | | Males | | | | Females | | | |
|---|---|---|---|---|---|---|---|---|---|---|---|---|---|
| | | c-ALL[b] | Infant | OR[c] | 95% CI | c-ALL | Infant | OR | 95% CI | c-ALL | Infant | OR | 95% CI |
| *01 | *0101/*0104 | 18.3[d] | 9.0 | 2.27 | 1.12–4.52 | 21.8 | 6.4 | 4.06 | 1.42–10.17 | 11.4 | 11.3 | 1.01 | 0.35–3.10 |
| | *0102 | 11.7 | 15.4 | 0.72 | 0.37–1.47 | 9.0 | 17.9 | 0.45 | 0.19–1.17 | 15.9 | 12.5 | 1.32 | 0.5–3.62 |
| | *0103 | 8.3 | 5.1 | 1.68 | 0.67–4.14 | 7.7 | 5.1 | 1.54 | 0.45–4.89 | 9.1 | 5.0 | 1.90 | 0.52–6.90 |
| | *0102/*0103 | 2.5 | 0.6 | 3.97 | 0.56–17.14 | 2.6 | 0 | – | – | 2.3 | 1.3 | 1.83 | 0.25–13.43 |
| | combined *01 | 40.8[e] | 30.1 | 1.60 | 0.97–2.62 | 41.0 | 29.5 | 1.66 | 0.86–3.17 | 38.6 | 30.0 | 1.46 | 0.69–3.13 |
| *02 | *0201 | 12.5[f] | 21.2 | 0.53 | 0.28–1.04 | 11.5 | 24.4 | 0.40 | 0.18–0.96 | 13.6 | 17.5 | 0.74 | 0.29–2.06 |
| *03 | combined *03 | 22.5[g] | 15.4 | 1.59 | 0.87–2.90 | 23.1 | 10.3 | 2.62 | 1.07–6.02 | 20.5 | 20.0 | 1.02 | 0.43–2.52 |
| *04 | *0401 | 2.5 | 1.3 | 1.97 | 0.4–8.38 | 0 | 2.6 | 0 | – | 6.8 | 0 | – | – |
| | *0401/0601 | 0.8 | 0 | – | – | 1.3 | 0 | – | – | 0 | 0 | – | – |
| | *05 | 20.8 | 31.4 | 0.57 | 0.34–1.0 | 20.5 | 30.8 | 0.58 | 0.29–1.20 | 20.5 | 31.3 | 0.56 | 0.25–1.35 |
| | *0601 | 0 | 0.6 | 0 | 0.04–4.8 | 0 | 0 | 0 | – | 0 | 1.3 | – | 0.05–6.75 |
| | combined *04 | 24.2 | 33.3 | 0.63 | 0.38–1.09 | 21.8 | 33.3 | 0.55 | 0.28–1.14 | 27.3 | 32.5 | 0.77 | 0.36–1.74 |
| n[h]= | | 60 | 78 | | | 38 | 38 | | | 22 | 40 | | |

[a]*DQA1*0101* and **0104* were not distinguished with the SSO probes used here. Heterozygotes with *DQA1*0102/*0103* and **0401/*0601* could not be distinguished. Combined **03* alleles include **03011* and **0302*. [b]Results are allele frequencies (%). [c]OR, odds ratios; 95% CI, 95% confidence interval. [d]*DQA1*0101/*0104*: total ALL vs total infant control subjects: two-sided Fisher's $P = 0.03$. Male c-ALL vs male infant control subjects: two-sided Fisher's $P = 0.01$. [e]Combined *DQA1*01* alleles: total c-ALL vs total control subjects: two-sided Fisher's $P = 0.08$. Male c-ALL vs male control subjects: two-sided Fisher's $P = 0.18$. [f]*DQA1*0201*: total c-ALL vs total control subjects: two-sided Fisher's $P = 0.08$. Male c-ALL vs male control subjects: two-sided Fisher's $P = 0.05$. [g]Combined *DQA1*03* alleles: total c-ALL vs total control subjects: two-sided Fisher's $P = 0.17$. Male c-ALL vs male control subjects: two-sided Fisher's $P = 0.05$. [h]*n*, number of subjects in each group.

### Data analysis

Differences in patient and control allele frequencies are expressed as odds ratios (OR) (Altman, 1991) together with 95% confidence intervals (CI) derived using Miettinens method (Breslow and Day, 1980). Patient and control *DQA1* allele frequencies were also compared using 2 × 2 analysis, and tested for significance by two-sided Fisher's exact tests. DQA1 and DQB1 exon 2 polymorphic amino acid frequencies (see Marsh) were compared in patients and control subjects using ORs and 2 × 2 tests. Observed and expected heterozygosity was compared using allelic diversity (*h*) (Nei and Roychoudhury, 1974). Sample size (i.e. statistical power) calculations were performed using nQuery Advisor release 2.0 (Statistical Solutions, Cork, Ireland).

### RESULTS

### Study group

The patients consisted of a prospective series of 38 boys and 22 girls with c-ALL aged between 1.6 and 12.9 years, with a mean age at diagnosis of 5 years 3 months, and a median of 4 years 4 months. There was a 26% excess of male to female patients, giving an M:F of 1.73:1. The mean (median) age of the male patients was 4 years 8 months (4 years 3 months), compared with the mean (median) age of the female patients of 6 years 2 months (4 years 10 months).

### *HLA-DQA1* alleles in c-ALL

All *DQA1* alleles detected in the c-ALL patients were also found in the infant control subjects. Analysis of heterozygosity (*h*) showed no significant difference between observed and expected values for c-ALL (obs., 71.%; exp., 66.4%, $P = 0.22$) and infant control subjects (obs., 66.7%; exp. 75.5%; $P = 0.22$) or between c-ALL and control subjects.

Table 1 shows that *DQA1*01* was more frequent in patients than control subjects (OR = 1.60; 95% CI: 0.97–2.62), mainly because of *DQA1*0101/*0104* (OR = 2.27; 1.12–4.52). There was also an increase in the *DQA1*03* (OR = 1.59; 0.87–2.90) and a deficit in *DQA1*0201* (OR = 0.53; 0.28–1.04).

Male c-ALL had a significantly higher frequency of *DQA1*0101/*0104* (OR = 4.06; 1.42–10.17) (Table 1), a higher frequency of *DQA1*03* (OR = 2.62; 1.07–6.02) and a deficit of *DQA1*0201* (OR = 0.40; 0.18–0.96) than male control subjects. Apart from a small deficit in *DQA1*0201*, there were no differences between female c-ALL and female control subjects.

### *DQA1* and *DQB1* alleles in c-ALL

Patients and control subjects were classified according to whether they typed for both *DQB1*0501* and *DQA1*0101/*0104*. Table 2 shows that the greatest difference was between male c-ALL and male control subjects (OR = 3.73; 1.19–10.3). This was absent in girls with c-ALL, and in four other pairs of *DQA1,DQB1* alleles known to be in linkage disequilibrium.

### HLA-DQ polymorphic amino acids

We previously found that c-ALL was associated with the *absence* of *DQB1* alleles coding for aspartic acid (Asp) at position 57 of DQB1 (DQβ1Asp57–; Dearden et al, 1996). As DQα1Arg52+, DQβ1Asp57– is a susceptibility haplotype in juvenile insulin-dependent diabetes (IDDM; Tosi et al, 1994), we analysed its frequency in c-ALL. We found no association with c-ALL (data not shown). Further analysis revealed an increased frequency of serine at position 52 (αSer52) of DQA1, and valine at position 57 (βVal57) of DQB1 (i.e. αSer52,βVal57) in c-ALL (Table 3; OR = 2.34; 1.07–49.7). This was confined to boys (OR = 4.18; 1.41–11.03) and was absent from girls with c-ALL (OR = 0.83; 0.25–3.0).

© Cancer Research Campaign 1998

**Table 2** Frequency of co-occurring *DQA1* and *DQB1* alleles in childhood common ALL

| *DQA1, DQB1*[a] alleles | Total | | | | Male | | | | Female | | | |
|---|---|---|---|---|---|---|---|---|---|---|---|---|
| | c-ALL | Infant | OR[c] | 95% CI | c-ALL | Infant | OR | 95% CI | c-ALL | Infant | OR | 95% CI |
| **0101,*0501* | 30.0[b] | 17.3 | 2.04[d] | 0.91–4.45 | 36.8 | 13.5 | 3.73 | 1.19–10.3 | 18.2 | 21.1 | 0.83 | 0.25–3.0 |
| **0102,*0602* | 11.7 | 16.0 | 0.69 | 0.27–1.85 | 5.3 | 16.2 | 0.28 | 0.08–1.39 | 22.7 | 15.8 | 1.56 | 0.46–5.39 |
| **03,*0302* | 11.7 | 12.0 | 0.96 | 0.36–2.66 | 13.2 | 10.8 | 1.25 | 0.34–4.39 | 9.1 | 13.2 | 0.66 | 0.17–3.29 |
| **0501,*0201* | 23.3 | 40.0 | 0.45[e] | 0.22–0.97 | 23.7 | 37.8 | 0.51 | 0.2–1.37 | 22.7 | 42.1 | 0.40 | 0.14–1.31 |
| **0501,*0301* | 20.0 | 16.0 | 1.31 | 0.56–3.08 | 18.4 | 16.2 | 1.16 | 0.37–3.56 | 22.7 | 15.8 | 1.56 | 0.46–5.39 |

[a]Co-occurring *DQA1* and *DQB1* alleles. [b]Figures are % allele frequencies. [c]OR, odds ratios; 95% CI, 95% confidence intervals. [d]*DQA1*0101/*0104 DQB1*0501*: total c-ALL vs total control subjects: two-sided Fisher's $P = 0.12$. Male c-ALL vs male control subjects: two-sided Fisher's $P = 0.03$. Female c-ALL vs female control subjects, not significant. [e]*DQA1*0501,DQB1*0201*: total c-ALL vs total control subjects: two-sided Fisher's $P = 0.06$. Male c-ALL vs male control subjects, and female c-ALL vs female control subjects, not significant.

**Table 3**: Frequency of c-ALL patients and control subjects with alleles coding serine at position 52 of *DQA1* and valine at position 57 of *DQB1*

| DQα1, DQβ1 heterodimers[a] | Total | | | | Males | | | | Females | | | |
|---|---|---|---|---|---|---|---|---|---|---|---|---|
| | c-ALL | Infants | OR[c] | 95% CI | c-ALL | Infants | OR | 95% CI | c-ALL | Infants | OR | 95% CI |
| αSer52+,βVal57+ | 35.0[b] | 18.7 | 2.34[d] | 1.07–4.97 | 44.7[c] | 16.2 | 4.18 | 1.41–11.03 | 18.2[c] | 21.1 | 0.83 | 0.25–3.00 |
| αSer52+,βVal57− | 51.7 | 49.3 | 1.09 | 0.68–2.71 | 44.7 | 54.1 | 0.68 | 0.29–1.68 | 63.6 | 44.7 | 2.16 | 0.74–5.90 |
| αSer52−,βVal57+ | 0 | 0 | – | – | 0 | 0 | – | – | 0 | 0 | – | – |
| αSer52−,βVal57− | 13.3 | 32.0 | 0.32 | 0.15–0.80 | 10.5 | 29.7 | 0.27 | 0.09–0.96 | 18.2 | 34.2 | 0.42 | 0.14–1.50 |
| $n^e$ = | 60 | 75 | | | 38 | 37 | | | 22 | 38 | | |

[a]DQα1 serine52 is coded by *DQA1*0101/0104,*0102,*0103*; DQβ1 valine57 is coded by *DQB1*0501, *0604–6, 8* and *9*. Combinations of amino acids are those where the specific codon is either present (+) or absent (–); (i.e. some other codon is present). [b]Figures are % frequency of patients and control subjects with each DQα1,DQβ1 heterodimer. [c]OR, odds ratios; 95% CI, 95% confidence intervals. [d]DQα1Ser52+, DQβ1Val57+: total c-ALL vs total infant control subjects: two-sided Fisher's $P = 0.05$. Male c-ALL vs male control subjects: two-sided Fisher's $P = 0.01$. Female c-ALL vs female control subjects: two-sided Fisher's $P = 1.06$. [e]$n$ = total number of subjects (i.e. c-ALL or control subjects) in each group (total, male or female).

The significance of these associations disappears following correction for the number of alleles tested. We therefore performed simulations based on allele and haplotype frequencies in the present study to estimate case and control sample sizes required to obtain statistical significance. We assumed 90% power to detect a $P = 0.005$ in a two-sided test, before correction for the number of alleles in equal numbers of cases and control subjects. For *DQA1*0101/*0104*, the total number of patients and control subjects is 475, and for boys it is 181. For *DQA1*0101/*0104,DQB1*0501* haplotypes, total patient and control series require 388 in each group whereas boys require 122 patients and controls.

### DISCUSSION

Evidence suggesting that the same HLA class II polymorphic sequences contribute both to the binding of antigenic peptides and disease susceptibility (Hammer et al, 1995; Kwok et al, 1996) suggests that an *HLA-DQA1,DQB1* haplotype association with childhood c-ALL could be construed as evidence of an infectious aetiology. In this study we found an increased frequency of *DQA1*0101/*0104*, and a deficit of *DQA1*0201* in c-ALL, suggesting roles in susceptibility and resistance to c-ALL respectively. Further analysis showed that *DQA1*0101/*0104* was increased in boys but not girls with c-ALL, a finding which would not have been expected by chance alone. Analysis of patients and control subjects classified by the presence or absence of *DQA1*0101/*0104 and DQB1*0501* showed that an increase in this 'haplotype' in patients was confined to boys. This haplotype (and certain others) encodes DQαSer52 and DQβVal57. Both amino acids were increased in c-ALL, but this was also confined to male patients. These results need to be treated with caution as they were not corrected for the number of alleles. However, we used the results to simulate the number of cases and control subjects required to repeat the study in an independent series.

The patients in this study were an unselected series with c-ALL, in which 63% were boys and 37% were girls (M:F 1.73:1). Analysis of 199 cases of childhood c-ALL in the Manchester Children's Tumour Registry (MCTR) for 1983–94 showed 118 boys and 81 girls (M:F 1.5:1), suggesting that the male excess was not due to chance. McKinney et al (1993) found no difference in the rate of c-ALL in boys and girls aged 1–9 in a UK study, but Buckley et al (1994) reported an M:F of 1.2:1 in 312 cases of c-ALL in a US study. We found no evidence that inclusion of only verified c-ALL patients, and exclusion of unclassified ALL favoured boys over girls. There was no difference in the age or gender of patients donating and not donating blood samples to the study.

The *DQA1* SSO probes used here define polymorphisms confined to exon 2 but do not distinguish between *DQA1*0101* and **0104*. These alleles differ for single base substitutions in codons 2 (exon 1) and 199 (exon 4) (Yasunaga et al, 1996). It remains to be seen whether the difference between **0101* and **0104* has any influence on susceptibility to c-ALL.

Our results contrast with Dorak et al (1995) who used a restriction fragment length polymorphism (RFLP)-based method to type *DQA1* alleles in childhood ALL. They found no increase in *DQA1-1A* in ALL, nor any difference between male and female patients

© *Cancer Research Campaign 1998*

typing for this allele. However, they found a significant increase in allele 3 in male compared with female ALL patients. We found an increase in *DQA1*03*, which was confined to boys with c-ALL. However, there was no difference in patients and control subjects typing for both *DQA1*03* and *DQB1*0302*, which are in linkage disequilibrium (Imanishi et al, 1992). Furthermore, there was no increase in the frequency of *DQA1*03* homozygotes in c-ALL.

As DQA1 is tightly linked to *DQB1*, the *DQA1*0101/*0104* association with c-ALL could be explained by linkage disequilibrium with *DQB1*0501*. The present study confirms an increased frequency of both alleles in boys with c-ALL, but not of other *DQA1/DQB1* haplotypes. Our previous results showed a reduced frequency of aspartic acid at position 57 in c-ALL (Dearden et al, 1996), suggesting similarities with the DQβ1Asp57– motif associated with IDDM (Tosi et al, 1994). However, analysis of the IDDM susceptibility haplotype DQα1Arg52,DQβ1Asp57– showed no evidence of a role in c-ALL.

*HLA-DQA1*0101, 0102, 0103* and *0104* all code serine at position 52, and *DQB1*0501, 0604-06, 0608* and *0609* code valine at position 57 (see Marsh). However, we found that only one haplotype, *DQA1*0101/*0104-DQB1*0501*, predominated in c-ALL. Thirty per cent of c-ALLs compared with 17.3% of control subjects had this haplotype. Analysed by gender, 36.8% of male c-ALL compared with 13.5% male control subjects expressed DQα1Ser52,DQβ1Val57 heterodimers, but there was no difference between female patients and controls subjects.

Gene transfection studies by Kwok et al (1993) showed that the expression of *DQB1*0501*-encoded β-chains is facilitated by *DQA1*0101* α-chains and is influenced by amino acids coded at the 3′ end of *DQB1*, corresponding to positions 60 and 91 of the DQβ1 subunit. Furthermore, *DQA1*0101-DQB1*0501* is one of the most common *DQA1-DQB1* haplotypes in the UK population (Doherty et al, 1992) and the second most common haplotype in French, Danish and Spanish populations (Imanishi et al, 1992). The gene expression and population genetic data thus suggest that the *DQA1*0101,DQB1*0501* haplotype may have had a selective advantage, possibly by protecting against infectious diseases.

Our results suggesting that susceptibility to c-ALL is increased in boys with a common *DQA1-DQB1* haplotype could be explained by a greater contribution of DQα1Ser52/DQβ1Val57 peptide-binding motifs to the protection of boys against certain types of childhood infection. There is increased susceptibility of male children to infections (Washburn et al, 1965; Purtilo and Sullivan, 1979), which suggests that certain HLA haplotypes may counteract X-linked defects in immunity in boys (Immunological Reviews, 1994), by promoting the efficiency of antigen presentation. If confirmed, our results would imply that a common *DQA1-DQB1* haplotype that may increase *resistance* to infection in boys has an important influence on *susceptibility* to childhood c-ALL. This would be consistent with the predictions of the Greaves hypothesis (Greaves, 1988; Greaves and Alexander, 1993), and further suggests that the candidate infection involved in c-ALL exhibits low pathogenicity but strong immunogenicity.

### ACKNOWLEDGEMENTS

This work was supported by a grant from the Kay Kendall Leukaemia Fund to GMT. JMB, VB and OBE are supported by the Cancer Research Campaign.

### REFERENCES

Alexander FE (1992) Space-time clustering of childhood acute lymphoblastic leukaemia: indirect evidence for a transmissable agent. *Br J Cancer* **65**: 589–592

Alexander FE, McKinney PA, Ricketts TJ and Cartwright RA (1990) Community lifetstyle characteristics and risk of acute lymphoblastic leukaemia in children. *Lancet* **336**: 1461–1465

Altman DG (1991) *Practical Statistics for Medical Research*. Chapman & Hall: London

Badrinath P, Day NE and Stockton D (1997) Seasonality in the diagnosis of acute lymphocytic leukaemia. *Br J Cancer* **75**: 1711–1713

Breslow NE and Day NE (1980) *Statistical Methods in Cancer Research*, Vol. 1. *The analysis of case–control studies*. pp. 134–136. IARC: Lyon

Buckley JD, Buckley CM, Ruccione K, Sather HN, Waskerwitz MJ, Woods WG and Robison LL (1994) Epidemiological characterisitics of childhood acute lymphocytic leukaemia. Analysis by immunophenotype. *Leukemia* **8**: 856–864

Dearden SP, Taylor GM, Gokhale DA, Robinson MD, Thompson W, Ollier W, Binchy A, Birch JM, Stevens RF, Carr T and Bardsley WG (1996) Molecular analysis of *HLA-DQB1* alleles in childhood common acute lymphoblastic leukaemia. *Br J Cancer* **73**: 603–609

Doherty DG, Vaughan RW, Donaldson PT and Mowat AP (1992) HLA DQA, DQB, and DRB genotyping by oligonucleotide analysis: Distribution of alleles and haplotypes in British Caucasoids. *Human Immunol* **34**: 53–63

Dorak MT, Owen G, Galbraith I, Henderson N, Webb D, Mills KI, Darke C and Burnett AK (1995) Nature of HLA-associated prodisposition to childhood acute lymphoblastic leukemia. *Leukemia* **9**: 875–878

Greaves MF (1988) Speculations on the cause of childhood acute lymphoblastic leukemia. *Leukemia* **2**: 120–125

Greaves MF (1997) Aetiology of acute leukaemia. *Lancet* **349**: 344–349

Greaves MF and Alexander FE (1993) An infectious etiology for common acute lymphoblastic leukemia in childhood? *Leukemia* **7**: 349–360

Hammer J, Gallazzi F, Bono E, Karr RW, Guenot J, Valsasnini P, Nagy ZA and Sinigaglia F (1995) Peptide binding specificity of HLA-DR4 molecules: correlation with rheumatoid arthritis association. *J Exp Med* **181**: 1847–1855

Imanishi T, Akaza T, Kimura A, Tokunaga K and Gojobori T (1992) Allele and haplotype frequencies for HLA and complement loci in various ethnic groups. In *HLA 1991, Proceedings of the 11th International Histocompatibility Workshop and Conference*, Vol. 1, Tsuji K, Aizawa M and Sasazuki T (eds), pp. 1065–1220. Oxford Science Publications: Oxford, UK

Immunological reviews (1994) Genetic basis of primary immunodeficiencies. **138**: 5–221

Kinlen LJ (1995) Epidemiological evidence for an infective basis in childhood leukaemia. *Br J Cancer* **71**: 1–5

Kinlen LJ (1997) High contact paternal occupations, infection and childhood leukaemia: five studies of unusual population-mixing of adults. *Br J Cancer* **76**: 1539–1545

Kwok WW, Kovats S, Thurtle P and Nepom GT (1993) HLA-DQ allelic polymorphisms constrain patterns of class II heterodimer formation. *J Immunol* **150**: 2263–2272

Kwok WW, Domeier ME, Raymond FC, Byers P and Nepom GT (1996) Allele-specific motifs characterize HLA-DQ interactions with a diabetes-associated peptide derived from glutamic acid decarboxylase. *J Immunol* **156**: 2171–2177

Marsh SGE HLA class I and II sequence alignments. Anthony Nolan Research Institute Web site at http://www.lif.icnet.uk/axp/tia/marsh/anri.html

McKinney PA, Alexander FE, Cartwright RA, Scott CS and Staines A (1993) Acute lymphoblastic leukaemia incidence in the UK by immunophenotype. *Leukemia* **7**: 1630–1634

Nei M and Roychoudhury AK (1974) Sampling variances of heterozygosity and genetic distance. *Genetics* **76**: 379–390

Noreen H, Hors J, Rønningen KS, Busson M, Khalil I, Lepage V and Bosnes V (1992) HLA-DQA1 and -DQB1 polymorphism using polymerase chain reaction (PCR) oligotyping. In *HLA 1991, Proceedings of the 11th International Histocompatibility Workshop and Conference*, Vol. 1, Tsuji K, Aizawa M and Sasazuki T (eds), pp. 477–484. Oxford Science Publications: Oxford, UK

Purtilo DT and Sullivan JL (1979) Immunological bases for superior survival of females. *Am J Dis Child* **133**: 1251–1253

Tosi G, Brunelli S, Mantero G, Magalini AR, Soffiati M, Pinelli L, Tridente G and Accolla RS (1994) The complex interplay of the DQB1 and DQA1 loci in the generation of the susceptible and protective phenotype for insulin-dependent diabetes mellitus. *Mol Immunol* **31**: 429–437

© Cancer Research Campaign 1998

Van Steensel-Moll HA, Valkenburg HA and Zanen GE (1986) Childhood leukemia and infectious diseases in the first year of life: a register-based case–control study. *Am J Epidemiol* **124**: 590–594

Washburn TC, Medearis DN and Childs B (1965) Sex differences in susceptibility to infections. *Pediatrics* **35**: 57–64

Westerbeek RMC, Biair V, Eden OB, Kelsey AM, Stevens RF, Will AM, Taylor GM and Birch JM (1998) Seasonal variations in the onset of childhood leukaemia and lymphoma. *Br J Cancer* (in press)

Yasunaga S, Kimura A, Hamaguchi K, Rønningen KS and Sasazuki T (1996) Different contribution of HLA-DR and -DQ genes in susceptibility and resistance to insulin-dependent diabetes mellitus (IDDM). *Tissue Antigens* **47**: 37–48

© Cancer Research Campaign 1998


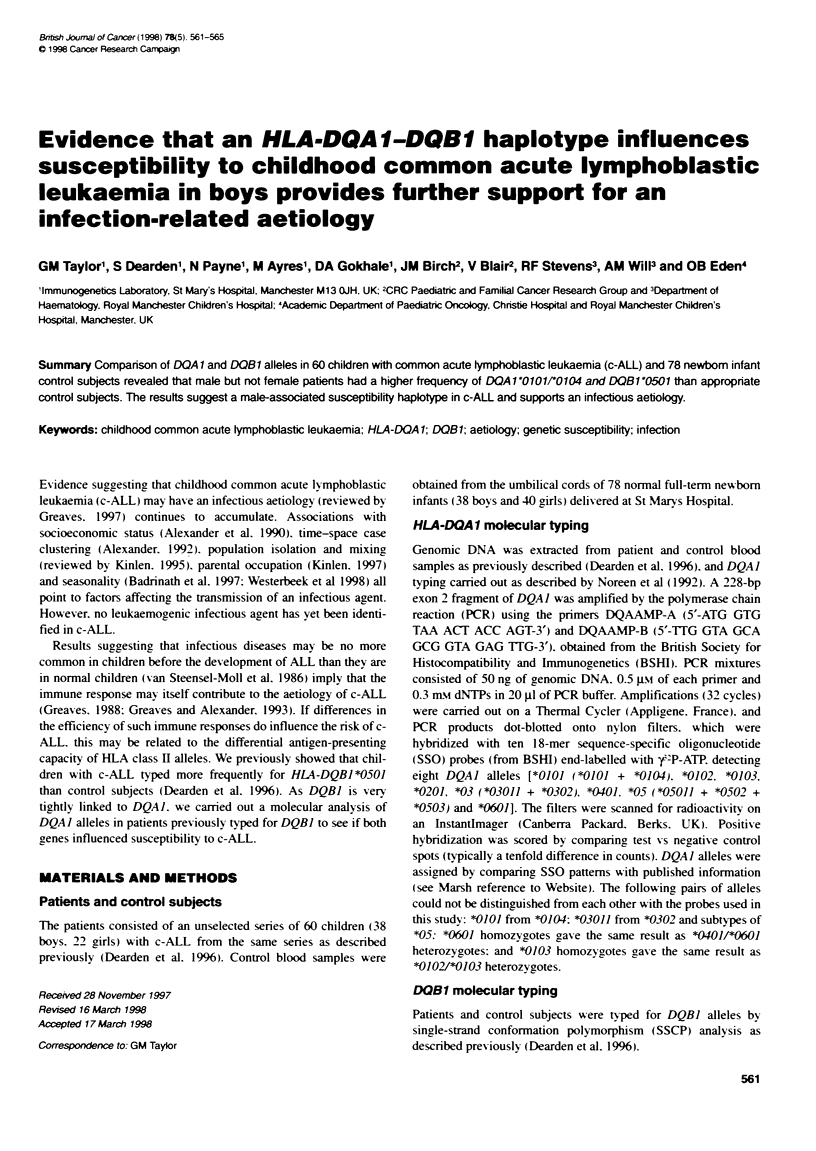

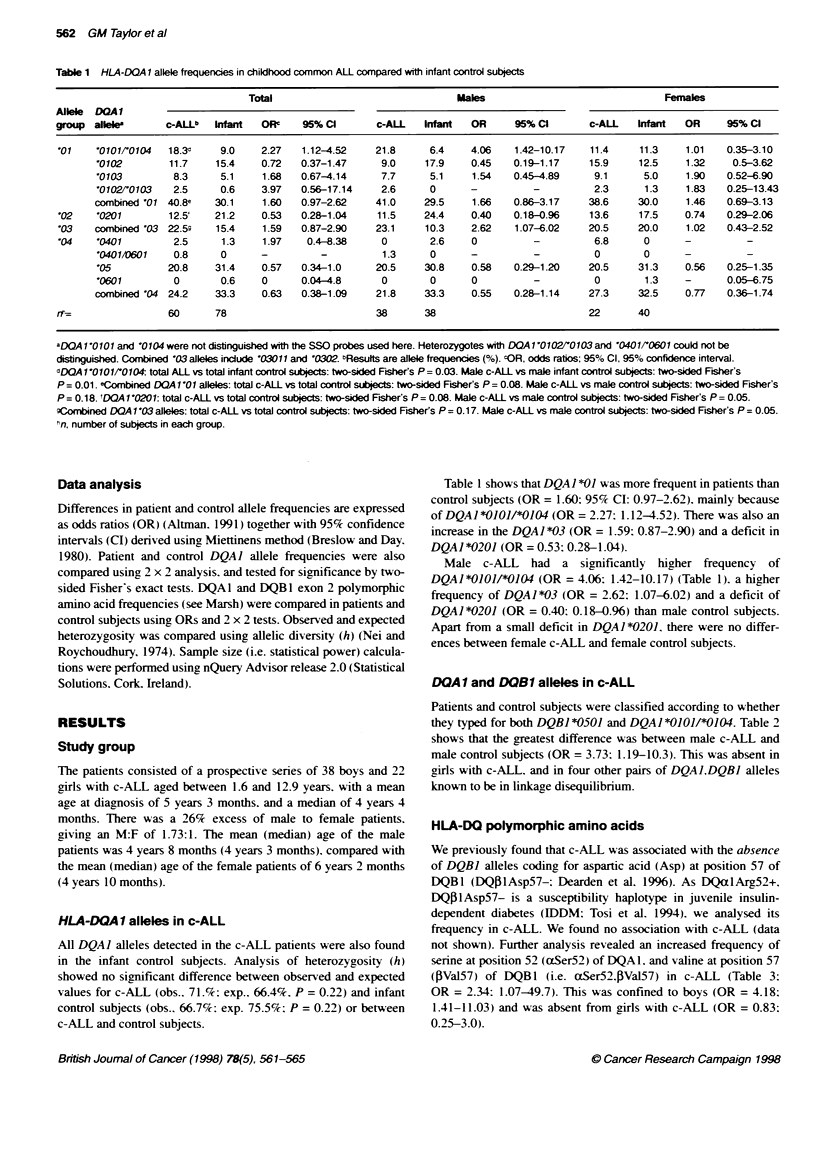

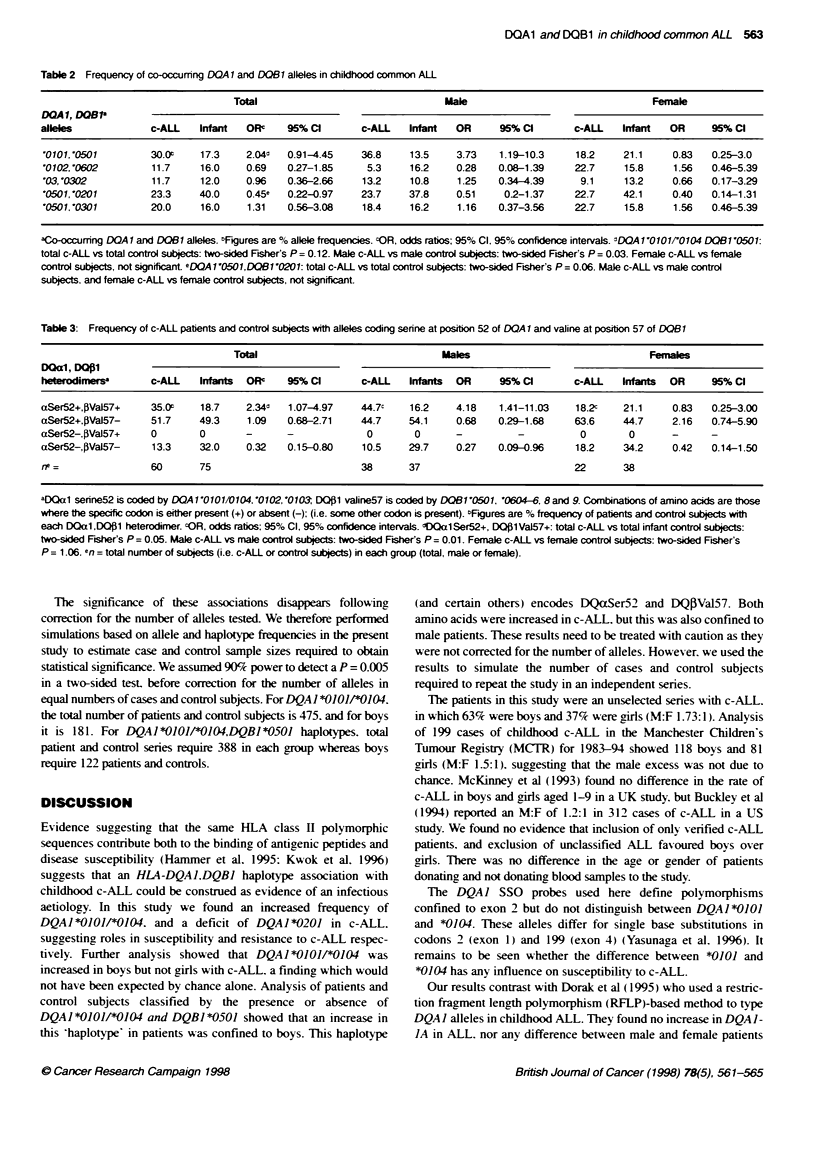

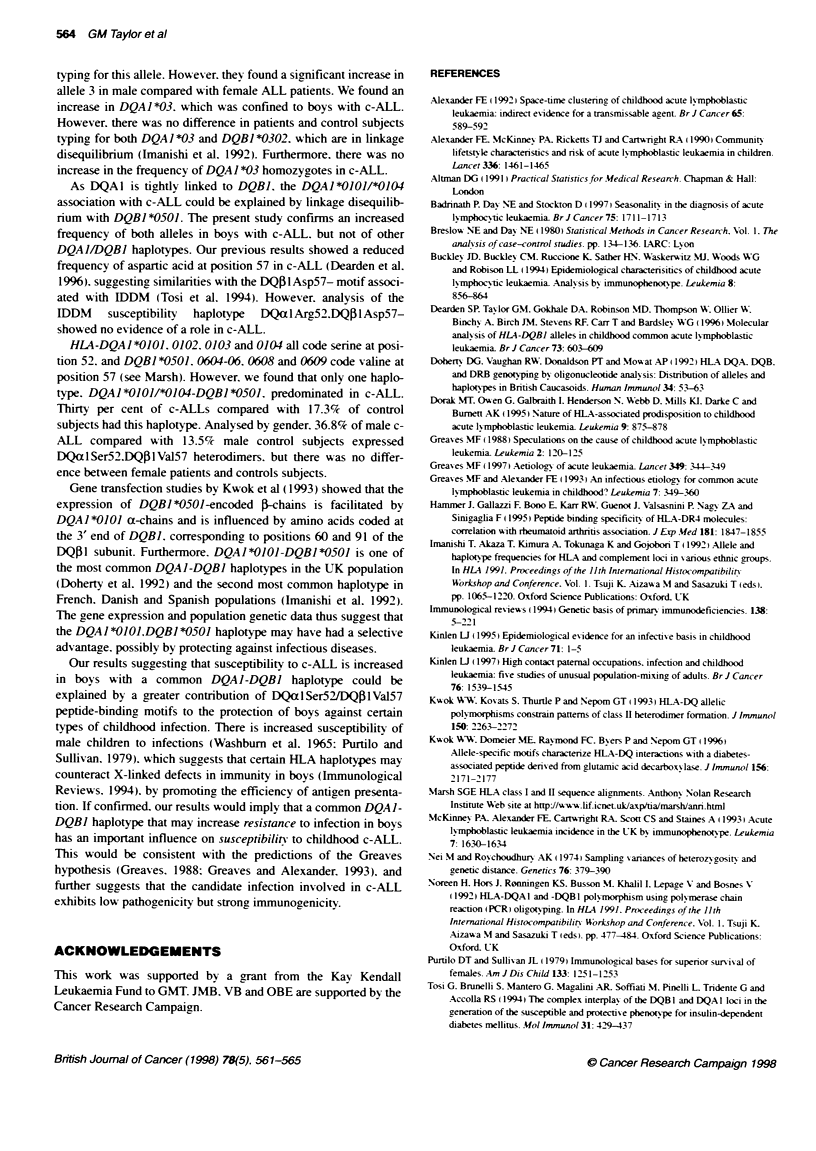

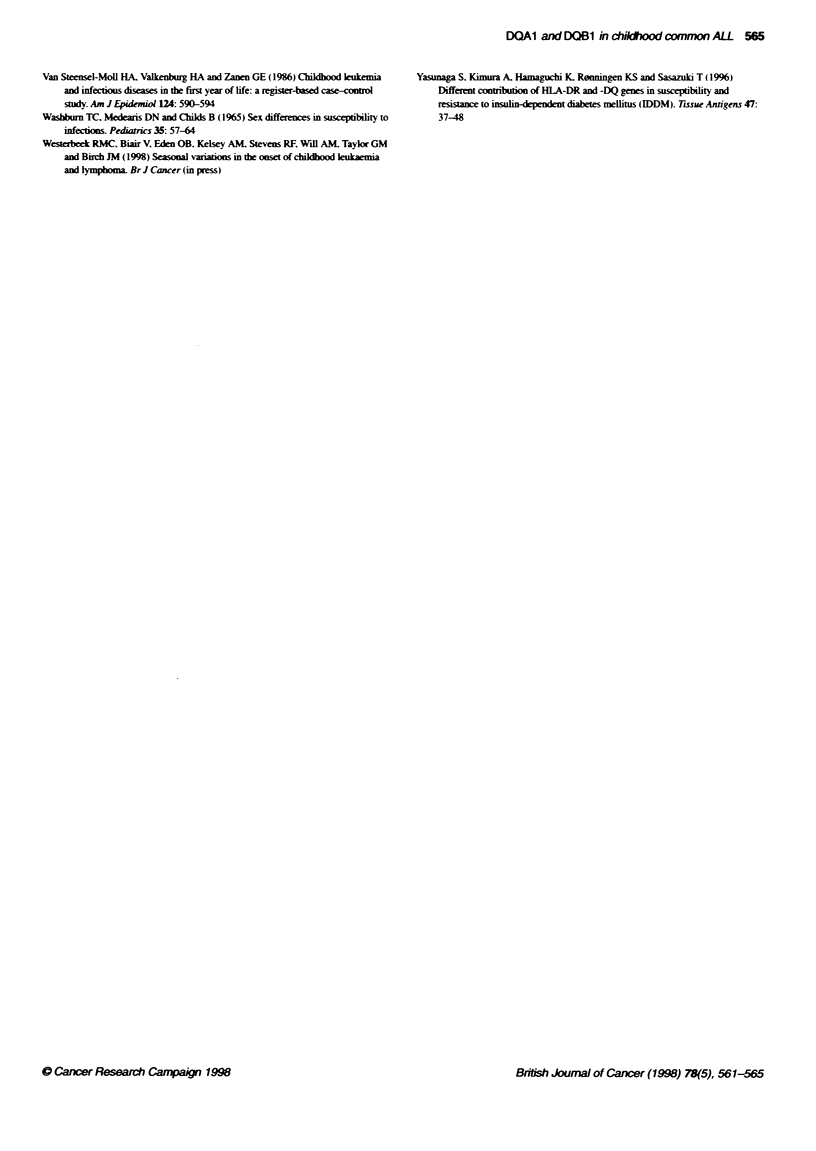


## References

[OCR_00398] Alexander F. E., Ricketts T. J., McKinney P. A., Cartwright R. A. (1990). Community lifestyle characteristics and risk of acute lymphoblastic leukaemia in children.. Lancet.

[OCR_00391] Alexander F. E. (1992). Space-time clustering of childhood acute lymphoblastic leukaemia: indirect evidence for a transmissible agent.. Br J Cancer.

[OCR_00405] Badrinath P., Day N. E., Stockton D. (1997). Seasonality in the diagnosis of acute lymphocytic leukaemia.. Br J Cancer.

[OCR_00416] Buckley J. D., Buckley C. M., Ruccione K., Sather H. N., Waskerwitz M. J., Woods W. G., Robison L. L. (1994). Epidemiological characteristics of childhood acute lymphocytic leukemia. Analysis by immunophenotype. The Childrens Cancer Group.. Leukemia.

[OCR_00419] Dearden S. P., Taylor G. M., Gokhale D. A., Robinson M. D., Thompson W., Ollier W., Binchy A., Birch J. M., Stevens R. F., Carr T. (1996). Molecular analysis of HLA-DQB1 alleles in childhood common acute lymphoblastic leukaemia.. Br J Cancer.

[OCR_00425] Doherty D. G., Vaughan R. W., Donaldson P. T., Mowat A. P. (1992). HLA DQA, DQB, and DRB genotyping by oligonucleotide analysis: distribution of alleles and haplotypes in British caucasoids.. Hum Immunol.

[OCR_00432] Dorak M. T., Owen G., Galbraith I., Henderson N., Webb D., Mills K. I., Darke C., Burnett A. K. (1995). Nature of HLA-associated predisposition to childhood acute lymphoblastic leukemia.. Leukemia.

[OCR_00439] Greaves M. F. (1997). Aetiology of acute leukaemia.. Lancet.

[OCR_00441] Greaves M. F., Alexander F. E. (1993). An infectious etiology for common acute lymphoblastic leukemia in childhood?. Leukemia.

[OCR_00435] Greaves M. F. (1988). Speculations on the cause of childhood acute lymphoblastic leukemia.. Leukemia.

[OCR_00462] Kinlen L. J. (1995). Epidemiological evidence for an infective basis in childhood leukaemia.. Br J Cancer.

[OCR_00468] Kinlen L. J. (1997). High-contact paternal occupations, infection and childhood leukaemia: five studies of unusual population-mixing of adults.. Br J Cancer.

[OCR_00471] Kwok W. W., Kovats S., Thurtle P., Nepom G. T. (1993). HLA-DQ allelic polymorphisms constrain patterns of class II heterodimer formation.. J Immunol.

[OCR_00489] McKinney P. A., Alexander F. E., Cartwright R. A., Scott C. S., Staines A. (1993). Acute lymphoblastic leukaemia incidence in the UK by immunophenotype.. Leukemia.

[OCR_00494] Nei M., Roychoudhury A. K. (1974). Sampling variances of heterozygosity and genetic distance.. Genetics.

[OCR_00512] Tosi G., Brunelli S., Mantero G., Magalini A. R., Soffiati M., Pinelli L., Tridente G., Accolla R. S. (1994). The complex interplay of the DQB1 and DQA1 loci in the generation of the susceptible and protective phenotype for insulin-dependent diabetes mellitus.. Mol Immunol.

[OCR_00524] WASHBURN T. C., MEDEARIS D. N., CHILDS B. (1965). SEX DIFFERENCES IN SUSCEPTIBILITY TO INFECTIONS.. Pediatrics.

[OCR_00535] Yasunaga S., Kimura A., Hamaguchi K., Ronningen K. S., Sasazuki T. (1996). Different contribution of HLA-DR and -DQ genes in susceptibility and resistance to insulin-dependent diabetes mellitus (IDDM).. Tissue Antigens.

[OCR_00517] van Steensel-Moll H. A., Valkenburg H. A., van Zanen G. E. (1986). Childhood leukemia and infectious diseases in the first year of life: a register-based case-control study.. Am J Epidemiol.

